# Chitin Nanofibrils Enabled Core–Shell Microcapsules of Alginate Hydrogel

**DOI:** 10.3390/nano13172470

**Published:** 2023-09-01

**Authors:** Thakur Sapkota, Bishnu Kumar Shrestha, Sita Shrestha, Narayan Bhattarai

**Affiliations:** 1 Department of Chemical, Biological, and Bioengineering, North Carolina A&T State University, Greensboro, NC 27411, USA; tsapkota@aggies.ncat.edu (T.S.); bishnuaampipal@hotmail.com (B.K.S.); sshrestha2@ncat.edu (S.S.); 2 Department of Applied Science and Technology, North Carolina A&T State University, Greensboro, NC 27411, USA

**Keywords:** chitin, nanofibrils, alginate, microcapsule, NIH/3T3 cells, in vitro, cytotoxicity

## Abstract

An engineered 3D architectural network of the biopolymeric hydrogel can mimic the native cell environment that promotes cell infiltration and growth. Among several bio-fabricated hydrogel structures, core–shell microcapsules inherit the potential of cell encapsulation to ensure the growth and transport of cells and cell metabolites. Herein, a co-axial electrostatic encapsulation strategy is used to create and encapsulate the cells into chitin nanofibrils integrated alginate hydrogel microcapsules. Three parameters that are critical in the electrostatic encapsulation process, hydrogel composition, flow rate, and voltage were optimized. The physicochemical characterization including structure, size, and stability of the core–shell microcapsules was analyzed by scanning electron microscope (SEM), FTIR, and mechanical tests. The cellular responses of the core–shell microcapsules were evaluated through in vitro cell studies by encapsulating NIH/3T3 fibroblast cells. Notably, the bioactive microcapsule showed that the cell viability was found excellent for more than 2 weeks. Thus, the results of this core–shell microcapsule showed a promising approach to creating 3D hydrogel networks suitable for different biomedical applications such as in vitro tissue models for toxicity studies, wound healing, and tissue repair.

## 1. Introduction

Engineering a customized pattern of cellular microenvironments helps to recapitulate native cell niches to facilitate tissue growth and repair. Such an engineered microenvironment can restore the features of the native extracellular matrix (ECM) within the inherent power of physicochemical modification, immunotherapy, controlled microstructure, and cell protection from external factors [1]. Attributing to the microscopic stability and structural integrity, the microsphere- or microcapsule-based three-dimensional (3D) modality has elicited much attention to validate the proof of concept for translational studies and therapeutic agents [2,3]. Various techniques including microfluidics, electrostatic droplet extrusion, 3D bioprinting, and so forth have been applied to fabricate 3D microtissue constructs hydrogels such as in the form of spheroids and organoids [4,5]. The simultaneous addition or combination of cells and gradients of microenvironment materials in the form of tissue constructs leads to the effective spatial distribution of cells, nutrients, and growth factors. Microcapsules and spheroids have more advantages, particularly their better biomimetic ex vivo model, cell transplantation, and controlled drug delivery [2,6]. The cells that are confined within the microspheres can interact directly between the cells and modulate the microenvironment providing geometrical and biological structures of native tissue [7]. Consequently, the cell encapsulation into microspheres/spheroids influences cell-to-cell communication, cell–ECM interaction, ECM deposition, diffusion, and regulation, along with cell proliferation and differentiation. In light of the structure and function, cells growing in the 3D microspheres model exist as a more ideal and superior strategy over the 2D model that spontaneously fails to complete interactions of monolayer cells–ECM microenvironment, which reflects uncertainty for creating in vivo-like microenvironments [8].

Recent advancements in bioengineering intend to grow biomimetic self-assembled microcapsules that exhibit metabolic profiles and physiological relevance to support vascularization, nutrient diffusion, and oxygen supplementation [7]. ECM-rich stem cell-based microspheres stimulate cells to secret anti-inflammatory substances and growth factors that help self-healing power and tissue regenerative ability [9]. However, excessive use of artificial ECM components in microcapsules changes the physiology of the cell microenvironment, referring to fibrosis, a failure bioengineering tissue construct that cannot be considered for long-term cell survival and promote tissue functions [10]. Polymeric soft nanomaterials such as dendrimers, lipids, nanoparticles, nanotubes, etc., are used extensively for microsphere hydrogels to encapsulate cells [11]. These nanomaterials exhibit favorable surface topography, porosity, and biocatalytic properties, as well as enhance their mechanical properties, which also influence the suppression of the generation of excessive reactive oxygen (ROS) as a function of antioxidants [12,13].

Among several nanomaterials, chitin fibrils characterized as fibrous nanomaterials with excellent biocompatible, and biodegradable properties have been used as synthetic ECM materials in tissue engineering applications [14]. However, their broad applications in tissue engineering are limited mostly because of their weak load-bearing properties. Physicochemical properties of the chitin fibrils, e.g., mechanical strength, flexibility, hydrophobicity, charge densities, conductivity, etc., can be improved by either blending or co-spinning with other synthetic polymers [15,16]. Chitin nanofibrils with PEGDE hybrid hydrogel promoted the proliferation of NIH3T3 activities and exhibited bactericidal properties [17]. The cell adhesive properties of chitin show hemostatic ability in wound sites, so can be used for wound healing applications [18]. Chitin microspheres are also used as drug carriers for controlled-release applications [19]. Additionally, the uses of chitin fibrils in biomedical applications are broadened after combining chitin with many other polysaccharides including chitosan, alginate, cellulose, hyaluronic acid, and so forth [20,21,22]. Among these polysaccharides, alginate is an attractive material for cell encapsulation and angiogenesis study due to its biodegradability, and easy gelation [22,23]. The alginate microspheres facilitate cell immobilization and protection that are capable of cell transplantation and wound healing applications. In our previous studies, we developed polymeric nanofiber-integrated (PNA) alginate microcapsules with HepG2 cells [24]. Those microcapsules successfully allowed cells to survive, grow, and proliferate without causing cytotoxicity to the cells. However, some limitations still exist for the real-time application of microcapsules in vivo for clinical applications. The uncontrolled swelling behavior, fast-release kinetic in the delivery system, and unpredictable dissolution behaviors, etc., raise concern for encapsulated cells for their broad applications, especially for cell therapy and tissue engineering applications [25].

To address the above challenges in developing bioactive hydrogel microcapsules with adequate structural integrity, mechanical stability, controlled degradation, and ability to recapitulate native ECM, we aim to develop biocompatible core–shell microcapsules (CSMs) of alginate and chitin composites. We have hypothesized that crosslinking between alginate and chitin nanofibrils through calcium bridging develops composite microcapsules with interconnected porous networks. The microcapsules with alginate/chitin in the core and outer layer with alginate via co-axial electrohydrodynamic methods could be used to encapsulate cells for their long-term application. The conceptual design has been employed by simultaneously seeding NIH/3T3 cells with CSMs as cell niches to support this hypothesis. This approach may be useful to achieve cells encapsulated with CSMs as a cell delivery vehicle in cell transplantation for improving the efficacy of therapeutic treatment.

## 2. Experimental Section

### 2.1. Preparation of Chitin Nanofibrils

Chitin flakes obtained from shrimp were purchased from Millipore Sigma, St. Louis, MO, USA, and were modified into nanofibers after 2% *w*/*v* was dispersed in sterile deionized (DI) water using nano-fibrillation process [26]. Briefly, 0.5 g of chitin was dispersed in 25 mL of DI water. The mixture was sonicated under N_2_ bubbling in ice bath for 3 h to disentangle the chitin flakes into nanofibrils followed by lyophilization for their morphological study (See Section 3). The chitin nanofibrils were sterilized under ultraviolet (UV) light before being used in fabrication process of microcapsules.

### 2.2. Fabrication of Core–Shell Microcapsules

The CSMs were fabricated by utilizing co-axial electrohydrodynamic atomization method. Briefly, medical grade alginate (PRONOVA UP MVG, Novamatrix, Industriveien 33 N-1337 Sandvika Norway) solution of 2% (*w*/*v*) was prepared in 1X HBSS buffer (Life Technology, Gaithersburg, MD, USA) under constant stirring for 12 h. The solution of alginate was mixed with chitin nanofibrils (detail in Section 2.1) and the mixture was vigorously stirred for 5 min. The core of microcapsule was developed under different conditions, such as change in composition of the alginate to chitin nanofibrils ratio (i.e., 90/10, 80/20, and 60/40) under different flow rates (i.e., 1, 3, and 6 mL/h) at three different voltages (i.e., 1 kV, 5 kV, and 8 kV) with alginate solution of 1% (*w*/*v*) for shell layer. As prepared alginate/chitin nanofibrils colloidal was loaded in syringe for core, set on vertically mounted fisher pump, and alginate on another syringe for shell was loaded on horizontal pump. Both syringes containing core and shell solution were connected to inner 24 (gauge) and outer (18 gauge) sections of a co-axial needle (ramé-hart instrument Co. Succasunna NJ 07876 USA), respectively. The distance between tip of the nozzle to gelation bath of calcium chloride (CaCl_2_) solution (0.15 M) in a beaker was maintained at about 45 mm. The core–shell microcapsules were developed at applied DC voltage with flow of the two solutions simultaneously and allowed to completely be gelled for 10 min. The microcapsules were rinsed with water to remove excessive calcium chloride before further characterization.

### 2.3. Optimization of Fabrication Parameters

The core of the microcapsule was constructed under optimal conditions. The composition of alginate/chitin ratio in core of microcapsule was prepared in different proportions of 90/10, 80/20, and 60/40 at constant voltage (5 kV) and flow rate (3 mL/h). We observed the structure of core of microcapsule. Based on the shape, size, and uniformity of the core microcapsules, the final composition of alginate/chitin ratio of 60/40 was kept constant for all other conditions (e.g., flow rate and applied voltage) for fabricating process of CSMs. Similarly, the core of the microcapsules was fabricated under different flow rates. The flow rate of homogenous mixture was allowed for (1, 3, and 6) mL/h for core and 15 mL/h for the shell at constant applied voltage of 5 kV. Furthermore, the core of microcapsules was prepared at different applied DC voltage (for example, 1, 5, and 8 kV) where the composition of core and flow rate were allowed to be fixed as optimal conditions. The microcapsules developed at optimal parameters were visualized under EVOS FL digital inverted microscope. The numbers of microcapsules, (*n* = 50) prepared at different compositions, flow rates, and applied voltages, separately were taken for the measurement of core size diameter using image J software (Version 1.41).

### 2.4. In Vitro Cell Culture

NIH/3T3, mouse fibroblast cell line (ATCC Cell Line Bank 1658, Manassas, VA, USA) were cultured in Dulbecco Modified Eagle Medium (DMEM) supplemented with 10% fetal bovine serum (FBS) and 1% penicillin/streptomycin (Gibco Thermo Fisher Scientific, Hampton, NH, USA). The cells were grown in tissue culture flasks at 37 °C in a 5% CO_2_ humidified environment. The fresh cell culture medium was replenished every second day of culture period. After 80% confluency, the cells were passaged by dissociation with trypsin–EDTA (Gibco, Thermo Fisher Scientific).

### 2.5. Cell Encapsulation in Core–Shell Microcapsules and Cytotoxicity Assay

**Figure 1:**
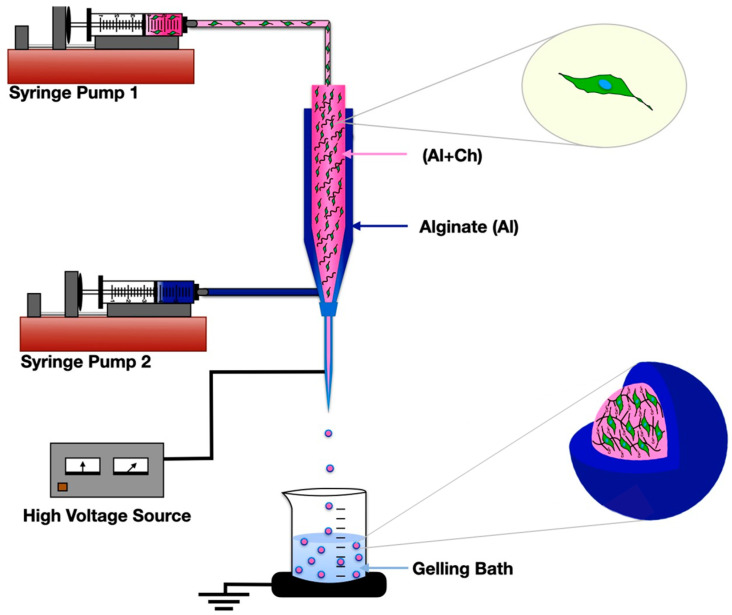
A schematic illustration showing design and fabrication of chitin nanofibrils enabled core–shell microcapsules of alginate hydrogel combined with NIH/3T3 fibroblasts.

The NIH/3T3 cells at a density of 1 × 10^6^/mL in cell culture media were suspended in equal volumes of alginate/chitin (80/20 and 60/40 *w*/*w*) separately, and the mixture was kept on a digital shaker for 10 min at 37 °C. As-prepared cell suspension was electrosprayed under the same condition as described in the fabrication of core–shell microcapsules at constant voltage of 5 kV and flow rate of 3 mL/h (Section 2.2). The overall fabrication procedure is depicted in the schematic representation (Figure 1). The cell-encapsulated core–shell microcapsules were immediately transferred into the Hanks’ balanced salt solution (1 X HBSS buffer, Thermo Fisher Scientific) The cell-encapsulated microcapsules were washed three times with HBSS at 5 min intervals. The encapsulated cells were transferred to 24-well plates with the complete cell culture mediums, DMEM, and cultured at 37 °C incubator for different time periods (i.e., 3, 6, and 9) days. The viability of the encapsulated NIH/3T3 cells was quantified by using AlamarBlue (AB) colorimetric assay. Briefly, the cell-encapsulated CSMs were rinsed with 1X PBS and de-gelled using sodium citrate (100 mM) solution. The cell suspension was then centrifuged to retrieve the cell pallets which were resuspended in fresh culture medium. Cell viability assay was performed after 4 h incubation of the collected cell medium in 96-well plates at 37 °C incubator. The culture well plate was protected from direct sunlight prior to the measurement. The absorbance of the AlamarBlue was recorded at 570 nm using 600 nm as a reference wavelength by a microplate reader (CLARIOstar Plus, BMG LABTECH Inc., Cary, NC, USA). In addition, cell viability percentage was monitored with trypan blue (TB) assay after de-gelling the cell encapsulated microcapsules and calculating the live or dead cells using Countess^TM^ Cell Counting Chamber Slide.

The viability of the encapsulated cells in the core–shell microspheres was examined with live and dead assay kits (Perkin Elmer LLC Via AOPI Staining Solution; Fisher Scientific, Hampton, NH, USA), in accordance with the company’s protocol. The live and dead cells stained green and red in color, respectively, were visualized under Olympus I×83 microscope incorporated with Olympus cell Sens Dimension software Version 3.2 (Olympus Corporation, Shinjuku, Tokyo, Japan).

Additionally, the morphology of the cells was studied by staining and visualizing the actin filaments and nuclei of the cells encapsulated in the microcapsules. At day 3 of the culture, the cells were washed with PBS and fixed with 4% paraformaldehyde (PFA, Thermo Scientific, Waltham, MA, USA) for 10 min. The fixed cells were permeabilized in 0.2% triton X-100 (Thermo Scientific) for 2 min at room temperature (RT). Then, the cells were blocked with 1% bovine serum albumin (BSA) for 30 min at RT. Subsequently, the cells were stained with Actin Green^TM^ 488 Readyprobes^TM^ reagent (Invitrogen, Thermo Fisher Scientific) for cytoplasm (20 min), and DAPI (4′6, -Diamidino-2-Phenylindole, Dihydrochloride; Invitrogen, Thermo Fisher Scientific), for nuclei (5 min), at RT in the dark condition. The fluorescence images were taken using Olympus I × 83 microscope (Olympus). Further, we evaluated the cell morphology of NIH3T3 seeded on 2D structure of the alginate/chitin (60/40 *w*/*w*) for comparative study with 3D cell encapsulated microcapsule. The alginate/chitin composite of 300 µL was pipetted on the center of 48-well plates and gelled with CaCl_2_ solution to develop 2D structure matrix. After the formation of 2D surface hydrogel, the cells were seeded at density of 1.5 × 10^4^/mL. After 3 days of cultivation, similar procedure was applied for staining the actin filaments and nuclei as described above for cell-encapsulated microcapsules.

### 2.6. Characterization

The morphology of the chitin nanofibrils and cross-section of lyophilized alginate/chitin composite was investigated using a scanning electron microscope (SEM; Hitachi, SU8000, Schaumburg, Illinois, USA) with an accelerating voltage of 10 kV. Zeta potential (ζ) of the materials including pure chitin, alginate, and alginate/chitin at different compositions were recorded with Malvern Zetasizer (ZEN3600; Malvern Instruments Inc. Westborough, MA, USA). The colloidal solution of pure chitin at 1 wt.% was further diluted in DI water (1/35, *v*/*v*) and 0.5 wt.% alginate solution was prepared in DI water separately before the ζ-potential measurements. For ζ-potential measurement of colloidal composite, 1 mL as prepared alginate solution was mixed with the chitin solution at different volumes of 3, 4, 5, and 6 mL, respectively. The chemical interactions between the different functional groups present in the hydrogel matrix were evaluated using ATR-FTIR Spectrometer (Agilent 670, Sant Clara, CA, USA). Attenuated total reflectance diamond was used to avoid the mixing of the sample with potassium bromide to gain the absorbance data. The spectra were recorded at the range of wavenumber 400–4000 cm^−1^. The microcapsules developed at various conditions and parameters were visualized under EVOS inverted light microscope (Hatfield, PA, USA), and size distributions of the core of microcapsules were analyzed using image J software (NIH, Bethesda, MD, USA). Furthermore, the mechanical strength of the core–shell microcapsules was measured using cell scale biomaterial testing: Micro Squisher (Micro Tester G2-MTG2-01, Waterloo, ON, Canada). The microspheres (*n* = 5) were immersed in different media (water, DMEM, and PBS) separately for up to 7 days at 37 °C before measurement. A constant force was applied through a circular tungsten micro-beam of diameter 0.5588 mm and length 58 mm. Single microcapsule was compressed to a maximum of 30% of their original diameter for 30 s and force–displacement curves and data were recorded automatically by the software associated with Micro-Tester-G2. Young modulus values of the core–shell microcapsules were computed as described in prior publications [26,27].

### 2.7. Statistical Analysis

All data were presented as mean ± S.D, and were analyzed using one-way analysis of variance (ANOVA) for significance. OriginPro software (Origin Lab 2023 version, Northampton, MA, USA) was used for data analysis. Post hoc Tukey’s test was performed with ANOVA for multiple comparisons. The *p*-values less than * *p* < 0.05 ** *p* < 0.01, and *** *p* < 0.001 were considered statistically significant.

## 3. Results and Discussion

### 3.1. Morphology and Structural Analysis

**Figure 2:**
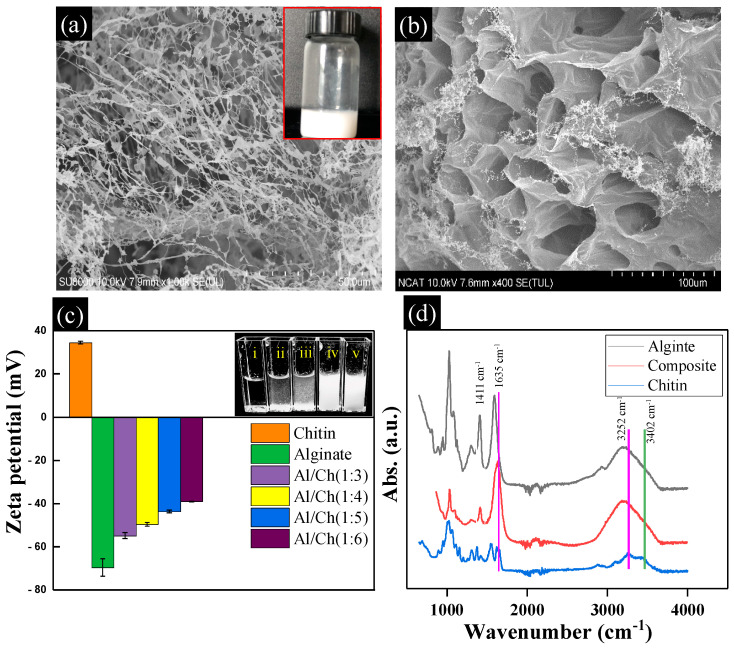
Characterization of chitin nanofibrils and alginate/chitin composites. (**a**,**b**) SEM images of chitin nanofibrils and cross-section of alginate/chitin nanofibril composites. The inset photograph of (**a**) shows the dispersion of chitin nanofibrils in DI water. (**c**) Zeta potential of alginate/chitin nanofibril mixture at different compositions. The inset figure of (**c**) represents optical images of the corresponding alginate/chitin mixture (*w*/*w*): i (100/0), ii (90/10), iii (80/20), iv (70/30), v (60/40). (**d**) Represents FT-IR spectra of alginate, chitin, and alginate/chitin composites.

The SEM image of as-prepared chitin nanofibrils and lyophilized composites of alginate/chitin are shown in Figure 2a,b. Non-woven assembly of an interconnected network of porous and spongy chitin nanofibrils is observed. The diameters of the fibrils are in wide ranges from ~130 nm to ~450 nm with the formation of irregular length and width. The length of the chitin nanofibrils was estimated higher than 500 µm. Nanofibrils with such a high aspect ratio can be considered as an ECM-like architecture that is found in living tissue microenvironments. The chitin fibrils as shown in the inset of Figure 2a reveal the complete disintegration of chitin flakes into nanofibrils using the ultrasonication/defibrillation process. Figure 2b shows the cross-sectional view of the alginate/chitin composites. The SEM image of the composite displayed a porous structure and the average pore sizes were measured at about ~50 µm. This porous framework of the composite exhibits suitable material properties, including mechanical properties, oxygen permeability, diffusion of nutrients, and migration or infiltration of cells, ensuring that the soft composite structure could be promising tissue constructs for biomedical applications [28].

Zeta potential value as depicted in Figure 2c illustrates the variation in surface charges of the alginate/chitin mixture with composition. The colloidal dispersion of pure chitin showed 34.66 mV whereas the alginate–chitin composite showed large values of ζ-potential. The ζ-potential value of pure alginate in the form of colloidal was measured at about −69.56 mV, indicating more negative charges in the surface of alginate due to the presence of a large number of -OH, -COOH functional groups. The ζ-potential of composites changed from −69.56 to −39.1 mV with the increased wt.% of chitin in composition (i.e., 1:3 to 1:6 (*v*/*v*). Chitin nanofibrils have high cationic charge density on the surface and the density can be with the changes in pH value [29]. The number of amino groups at low pH ionizes and increases the electrostatic repulsion between nanofibrils. Notably, the formation of the colloidal mixture was the aftermath of the electrostatic coupling of negative alginate with cationic-charged chitin that led to an increase in ζ-potential value. Obviously, the higher wt.% of chitin in the composite gradually increased the ζ-potential value. The photograph of alginate/chitin mixtures in the inset (Figure 2c) illustrates the dispersed particles in a stable stage due to electrostatic repulsion between clouds of positive charges in chitin nanofibrils. Figure 2d demonstrates the FTIR spectra of alginate, chitin, and alginate/chitin hydrogel composites. A comparison of these spectra at different peak positions reveals the chemical recognition by functional groups. The characteristic broad peaks of chitin nanofibrils at 3402 cm^−1^ and 3252 cm^−1^ assigned to O-H and N-H stretching bands, and other bands at 1740, 1661, and 1560 cm^−1^ correspond to the carboxyl, amide I, and amide II groups, respectively. All these absorption peaks and positions of chitin are consistent with the previously reported literature [30]. The existing two vibrational bands at 1411 cm^−1^ and 1590 cm^−1^ correspond to the symmetric and antisymmetric stretching bands of alginate [31]. The band at 3383 cm^−1^ confirms the hydroxyl groups assigned to the O-H stretching of alginate. In comparison, a slight shift in the peak position of the alginate/chitin composite to a higher wavenumber compared to individual polymers was recorded at 1635 cm^−1^, suggesting that the interaction between two polymers through the electrostatic interaction of amide I and COOH groups produced colloidal mixture, and also supported the increase in ζ-potential values as seen in Figure 2c. It is also noted that the less intensified vibrational band of pure alginate at 1411 cm^−1^ appeared in the composite at the same position confirming the homogeneity of the colloidal mixture of alginate with chitin nanofibrils.

**Figure 3:**
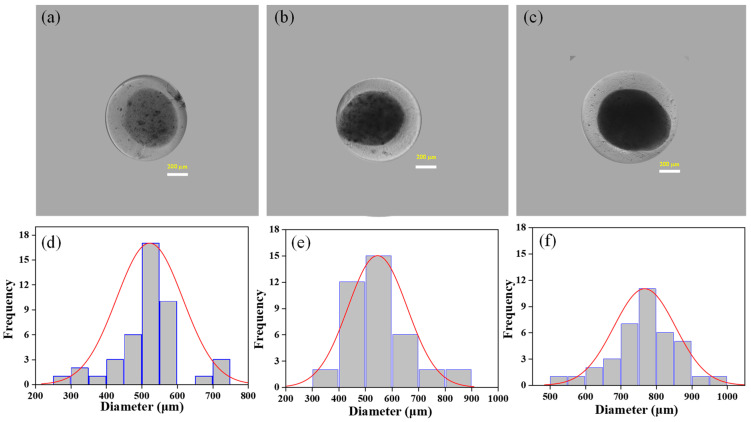
Morphology of core–shell microcapsules at different compositions. Top panel: Optical images of microcapsules with different ratios of alginate/chitin at the core, (**a**) 90/10, (**b**) 80/20, (**c**) 60/40. Shell composition was 1% *w*/*v* of alginate. Bottom panel: Size distribution of the core of the microspheres. The average diameters of the core are expressed quantitatively in frequency distribution, where (**d**–**f**) correspond to images (**a**–**c**), respectively.

Figure 3a–c shows photographs of the microcapsules in core–shell structure fabricated via co-axial electrospray of alginate/chitin colloids. The core of the microcapsule was alginate/chitin whereas the shell was alginate. The bright field micrographs of all core–shell microcapsules are relatively spherical as shown in Figure S1 of supplementary information (SI). The average diameters of the cores were found ~540 µm, ~630 µm, and ~725 µm, corresponding to alginate/chitin composition for 90/10, 80/20, and 60/40, respectively (Figure 3d–f). The average diameters of the fabricated cores increased with an increase in chitin wt.% ratio. It might be possible that the high charge density of higher polymeric concentration increases at the core of the microcapsules and raises the intramolecular repulsion and the viscosity, which tend to increase the size. Obviously, the dense core structure of the microsphere was observed due to the formation of a highly viscous composite with high chitin wt.%. Interestingly, the interior of the alginate/chitin (60/40) microsphere is more uniform and regular in size compared to others. The average diameters of the core–shell microcapsules (*n* = 50) were measured and found to be ~794, ~813, and ~950 µm, for composition (90/10), (80/20), and 60/40, respectively. However, the thickness of the shell did not show a noticeable difference. The average thickness of the shell of all the microspheres was about 150 µm with a smooth surface.

**Figure 4:**
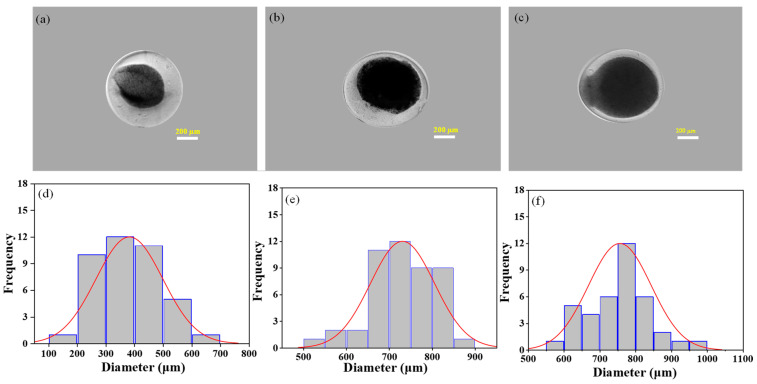
Morphology of core–shell microspheres at different flow rates. Top panel: Optical images of core–shell microspheres at different flow rates of core composition (**a**) 1 mL/h, (**b**) 3 mL/h, (**c**) 6 mL/h. Flow rate of shell composition and voltage were kept constant, 15 mL/h and 5 kV, respectively. Composition of alginate/chitin was fixed at 60/40. Bottom panel: Size distribution of core of the microspheres. The average diameters of the microspheres are expressed quantitatively in frequency distribution, where (**d**–**f**) correspond to images (**a**–**c**), respectively.

Figure 4 shows the photographs of core–shell alginate/chitin microcapsules (60/40) and frequency distribution of the core diameter of the CSMs at a different flow rate of the core solution. All the microcapsules are fine spherical shell structures. However, the shape and size of the core are varied with different flow rates at an applied voltage of 5 kV. Notably, the average diameter of the core increased with an increased flow rate revealing that at a higher flow rate, a large volume of solution is ejected from the needle forming large droplets of the composites. In the preparation, a flow rate of 3 mL/h produced a structurally intact spherical core with a mean diameter of 730 µm and a shell of alginate layer with a thickness of ~75 µm (Figure 4b). In contrast, the mean diameters of the cores were ~382 ± 119 µm and ~757 ± 87 µm at flow rates of 1 mL/h and 6 mL/h, respectively. It is also observed that the shape of the cores at 1 mL/h and 6 mL/h produced were more irregular with expanded and broken edges (Figure 4a,c and Figure S2 of SI). It is important to note that the weak surface boundary of microcapsules impairs the protection of cells in their in vitro and in vivo applications [32]. In cell encapsulation and cell therapy applications, such weak structures are less biotolerable and more susceptible to leaching out nutrients. Consequently, cell growth ability for long periods in microcapsules could be impeded, and leaking the cellular matrices from the microcapsules can induce inflammatory activity by host cells. It has been reported in our previous study that the size of the microcapsules from 300 to 500 µm provides better cytocompatibility with cellular spreading and prolonged proliferation of A549 cells [26]. Thus, microcapsules with regular spherical shapes and structural rigidity provide a promising approach for the design and synthesis of cell-laden microenvironments [33].

**Figure 5:**
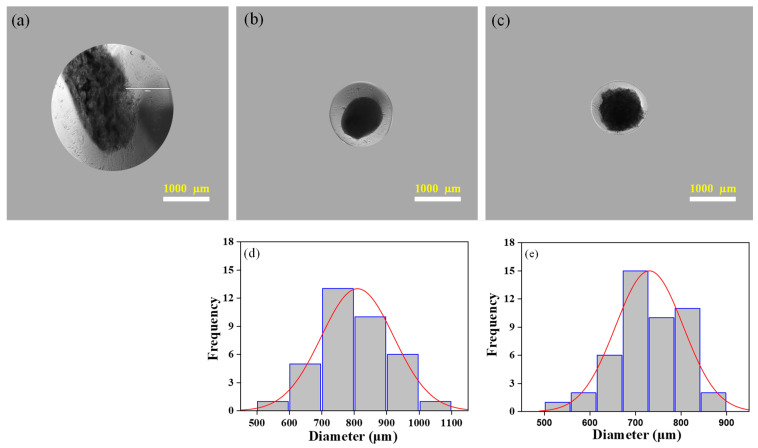
Morphology of core–shell microcapsules at different voltages. Top panel: Optical images of core–shell microcapsules at different voltages (**a**) 1 kV, (**b**) 5 kV, (**c**) 8 kV. Composition of alginate/chitin was fixed at 60/40 with a flow rate of3 mL/h. Bottom panel: Size distribution of core of the microspheres (*n* = 50). The average core diameters of the microcapsules are expressed quantitatively in frequency distribution, where (**d**,**e**) correspond to images (**b**,**c**), respectively.

Figure 5 shows the typical optical images of CSMs developed at different voltages while the alginate/chitin composition for core (60/40) and flow rate 3 mL/h remained fixed. At 1 kV, a regular spherical shape did not form. At low applied voltage, the effect of the electrostatic field is unable to form a stable Taylor cone, so the electrospray solution accumulates faster with irregular droplets (Figure 5a). Most of the microcapsules in the form of complete core–shell spherical geometry were produced at 5 kV (Figure 5b). The core diameters were measured in the ranges from 700 to 800 µm. At a high electric field, under the applied voltage of 8 kV, the absence of a Taylor cone resulted in the formation of jet spraying with relatively small diameter microcapsules, but the core diameters of the microcapsules were not measured remarkably differently (Figure 5c). Thus, the applied voltage at 5 kV was defined as the critical voltage in our work. At a critical voltage, the flow rate of the electrospray solution forms a meniscus at the tip of the needle that elongates to a calcium chloride bath with the formation of a Taylor cone for consistent droplets [34]. Figure S3 of SI also illustrates the spherical, regular, and uniform shape of CSMs prepared at 5 kV.

### 3.2. Mechanical Properties

**Figure 6:**
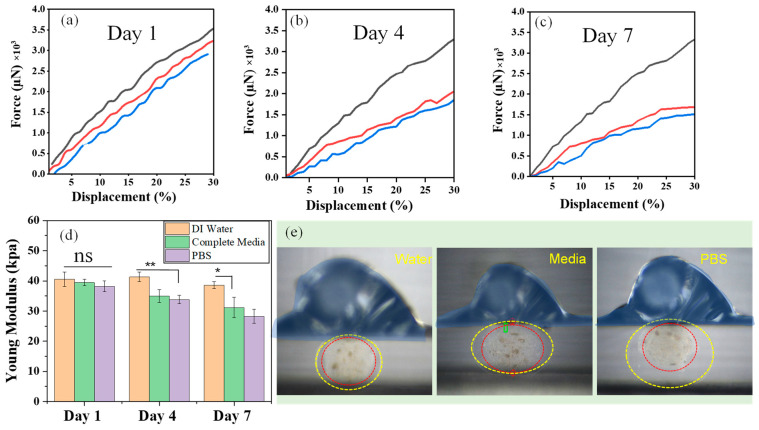
Mechanical properties of the core–shell microcapsules. Top panel: The force–displacement curves for core–shell microcapsules in different aqueous environments at different time periods. Alginate/chitin microcapsules with a composition of 60/40 were used in this study. (**a**–**c**) Typical force–displacement curves of the microcapsules incubated in DI water, DMEM cell culture media, and PBS for up to 7 days separately (black, red and blue colors represent force displacement curves measured in DI water, complete cell media and PBS). (**d**) The average Youngs modulus of the microcapsules (*n* = 5) incubated in the different aqueous media at different time periods (*p*-values less than * *p* < 0.05 and ** *p* < 0.01 were considered statistically significant). (**e**) Photographs showing the deformation of the microcapsules under compression test after 4 days of immersion in different mediums separately, where red and yellow dash circles indicate the core and shell of the microcapsules respectively. All of microcapsules were photographed at 3X magnification.

Figure 6a–c shows the force–displacement curves under compression test to verify the mechanical integrity of the core–shell microcapsules. The CSMs immersed in different media for various time periods showed distinct compressive stress. All the CSMs showed viscoelastic behavior at small deformation. The microcapsules treated in water withstand higher compressive strength of Ca. 3.5 × 10^3^ µN. This value is almost the same for all microcapsules treated in water for 7 days indicating the CSMs’ excellent structural stability in water. It can be observed that the microcapsules treated in cell culture media and PBS lost their mechanical strength further with longer immersion time. The microcapsules treated in cell media had compressive stress of ~2016.6 and 1683.3 µN at days 4 and 7, respectively. However, these values are higher compared to the values obtained while treated in PBS. Core–shell microcapsules also illustrate the loss of mechanical strength at different time periods in different media (Figure 6d). At day 1, the compressive Young moduli of the microcapsules treated in water, cell media, and PBS were 40.54 ± 2.34, 39.42 ± 0.05, and 38.18 ± 1.12 kPa, respectively. Notably, the value reduced significantly in cell media and PBS after 4 days. Importantly, the ionic concentration of the cell media and PBS has significant roles in lowering the Young moduli. Obviously, high cationic charge density in PBS takes part in ion-exchange reactions or the active sodium ions in PBS could displace the less reactive calcium from the Ca-crosslinked alginate microcapsules. Consequently, Young moduli of core–shell microcapsules in PBS are highly reduced. However, the outer layer of the microcapsule remains unaffected in pure water indicating the alginate hydrogel layer exhibits mechanical stability because of the non-ionic interactions. To assess cell encapsulation, the microcapsules should have adequate structural integrity and mechanical toughness where cell-integrated microcapsules can protect transplanted cells from external force exerted by host tissue during in vivo performance. The result indicates that the chitin nanofibrils enabled core–shell microspheres to have enhanced compressive moduli compared to other types of hydrogel constructs, e.g., silk-based hydrogel [35]. The optical micrographs as depicted in Figure 6e demonstrate the deformation of surface structure during compressive stress experiments. The expansion of the shell at a compression strain of 30% increase in the order of: water < cell media < PBS. The counter-ionic interactions between alginate and PBS weaken the interaction of alginate and chitin in the gel and as a result, the shell became easily swollen during compression.

### 3.3. Cytocompatibility

**Figure 7:**
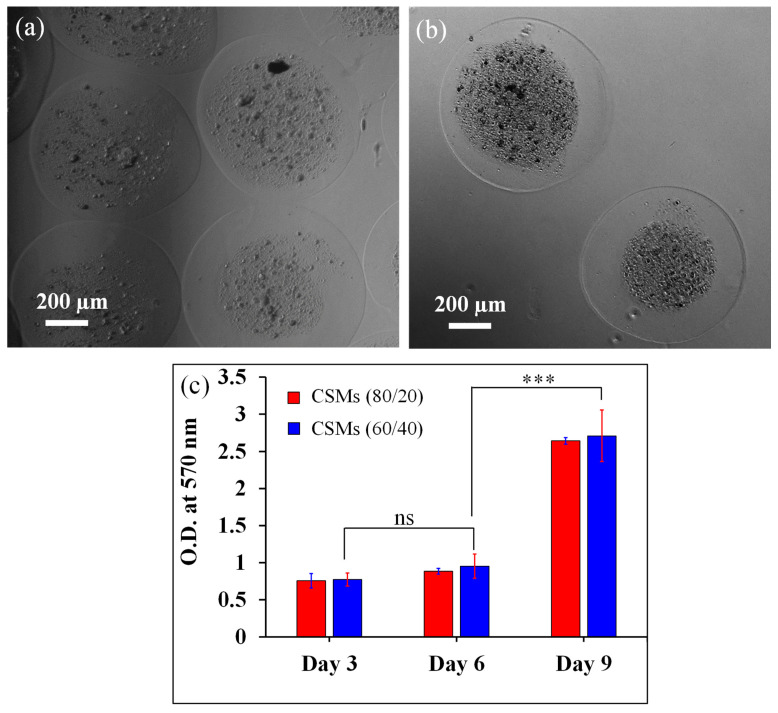
In vitro performance of microcapsules and cell viability study. (**a**) Microscopy images of NIH/3T3 encapsulated core–shell microcapsules with two different core compositions of alginate/chitin (**a**) 80/20, (**b**) 60/40. Cells were only mixed in core fluids. (**c**) Cytocompatibility evaluation of the core–shell microcapsules using AlamarBlue assays up to day 9. Statistical significance between the groups was followed by one-way ANOVA post hoc Tukey method. Data are represented as mean ± SD (*n* = 3; *** *p* < 0.001).

The core–shell microcapsules of alginate/chitin composite in the ratio; 80/20 and 60/40 were selected for cell encapsulation after the validation of structure, morphology, and physicochemical stability. The optical images of NIH/3T3 cells encapsulated CSMs after 6 days are shown in Figure 7a,b. Cell-encapsulated microcapsules of the composition of 60/40 showed a uniform shell (alginate) layer without surface polarization and rupture, suggesting that the composite can be a valuable cell delivery vehicle to assist cell transplantation for tissue regeneration in wound healing applications. The microcapsules with high wt.% of chitin nanofibrils in the core reinforce the mechanical integrity leading to providing a confined circular boundary that favors cell adhesion, cell–cell interaction, and proliferation. It seems that the uniform dispersion of chitin nanofibrils could generate an effective bridge at the composite interface, sufficient to avoid the premature fracture of the surface boundary. The synergistic effect of the shell layer and the chitin nanofibrils make the CSMs strong enough to protect the cell from the external environment directly. It is important that the cell-encapsulated microcapsules or spheroids with biologically stable coating improve the strong adhesion to the surrounding wet environment of host tissue for successful implantation [36]. We also evaluated the in vitro cell performance of cell-encapsulated alginate hydrogel alone microcapsules. We found the encapsulated cells escaped out of the microcapsules after certain days of culture and media replacements. We found a significant number of cells attached to the culture plate outside of the microcapsules at day 9 of the culture (see Supplementary Information, Figure S4). This is a critical issue with the use of alginate alone microcapsules for long-term culture. To overcome such an issue, we developed core–shell microcapsules of alginate/chitin.

Furthermore, the cytocompatibility of the CSMs was assessed after evaluating metabolic activity with AlamarBlue (Figure 7c). Both cell-encapsulated core–shell microcapsules supported enhanced metabolic activities over 9 days. The cellular metabolism of NIH/3T3 cells on each group of microcapsules determined the biocompatibility of the CSMs. The cell proliferation gradually enhanced for a longer incubation time of 3, 6, and 9 days, but the CSMs (80/20) showed less proliferation compared to CSMs (60/40). However, cellular metabolism on 3 and 6 days has no significant difference. In contrast, the cellular metabolism was significantly increased after 6 days (*** *p* < 0.001) as compared between each group. Beyond 6 days, the cell was completely adopted within microcapsules and began to perform its normal functions, for example, cell overgrowth, and self-stimulation on the immune response, and tend to differentiate [37]. The presence of high wt.% chitin interconnected nanofibril networks improve the mechanical stimulus and display abundant porosity that allows an easy percolation and supplement of water, protein, and growth factors to support cell growth and proliferation [38]. Further, the bioinspired nanofibrils in microcapsules exhibit interfacial toughness, and cell adhesive behavior, similar to cell instructive ECM-like features. Importantly, the cell surrounded by a thin layer of alginate further protects cells from destruction by external host tissue stress with immune protection, and appropriate permeability that may be beneficial for survivability of cells. In addition, the cell encapsulated by an alginate layer ensures the diffusion of protein, nutrient, and oxygen supply. Such dynamic structural features of microspheres or spheroids could assist the biophysiochemical activities continuously within host tissue environments.

**Figure 8:**
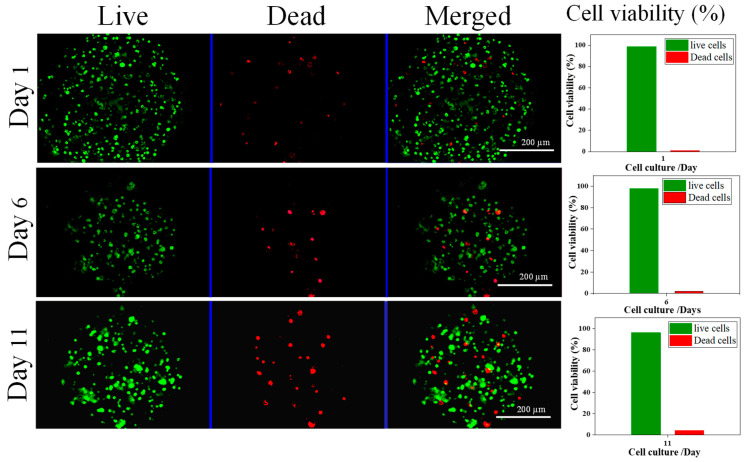
In vitro performance of microcapsules and cell viability study. Fluorescence microscopy images of the cell-encapsulated microcapsules representing live (stained green) and dead (stained red) NIH/3T3 fibroblasts. Cells were stained with Perkin Elmer LLC Via AOPI staining dye. Cells were uniformly distributed in the microcapsules. Scale bar = 200 µm. Far-right panel: The bar graphs represent the counted number of live and dead cells in the respective microcapsule’s fluorescent images shown in left panel (*n* = 10).

We evaluated the behavior of NIH/3T3 encapsulated in core–shell microcapsules (Figure 8). Live/dead staining images using fluorescence microscopy showed that over 95% of encapsulated live NIH/3T3 cells were visible after 11 days of culture suggesting that CSMs are an excellent biomaterial for cellular activity. Minimal cell death is observed in each cell cultured period, represented by fewer dead cells (i.e., stained with red fluorescence dye) compared to a higher number of live cells (i.e., stained with green fluorescence dye). The percentage of live cells is significantly higher than dead cells (quantitative data correspond to live and dead fluorescence images on different days (Figure 8, far right bar graphs). The cell viability decreased from 99% to 96% but no significant difference in viability was observed when the cultured period was extended to 11 days suggesting that the CSMs of (60/40) composition have low toxicity. It is well known that the composite has an advantage for cell interaction due to its low toxicity, hydrophilicity, mucoadhesive, and hemostatic properties for promoting wound healing functions [23]. Additionally, these composite polymers have the ability to stabilize generated reactive oxygen and reactive nitrogen species during cellular metabolism and effectively control excessive oxidative-stress- and nitrogen-stress-causing factors of cell death and apoptosis [39]. These results reveal that the composite promotes the proliferation of NIH/3T3 cells without notable toxicological effects.

**Figure 9:**
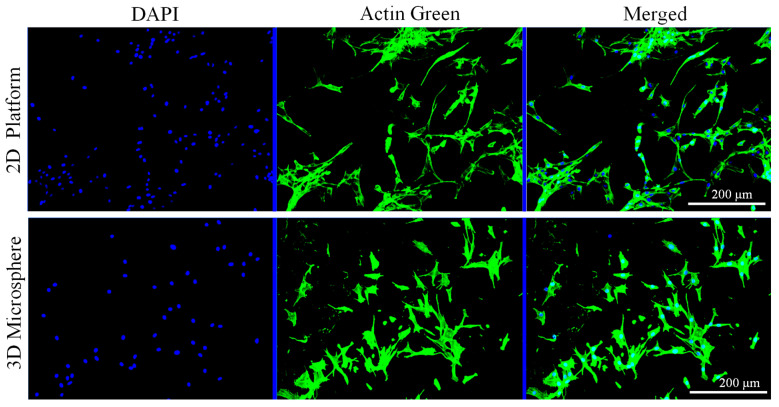
Comparative study of cell responses on two different alginate/chitin microenvironments (60/40). Top panel: NIH/3T3 fibroblasts cultured on the hydrogel surface (2D surface). Bottom panel: NIH/3T3 fibroblasts encapsulated in the core–shell microcapsule (3D micro-environment). Fluorescence microscopy images of the NIH/3T3 fibroblasts cultured in the two different environments were taken on day 3. Cytoskeletons were stained by actin green and counter-stained with DAPI (blue) for the nuclei. Scale bar = 200 µm.

In order to evaluate the effect of alginate/chitin composite on cell adhesion and proliferation, the fluorescence images of cells encapsulated in microcapsules were observed (Figure 9). It is important that the 3D microcapsules provide structural scaffolding with a realistic microenvironment where cellular mechanisms are performed as compared to the in vivo model. The cells cultured in the 3D microspheres are able to sense and respond to the signals from surrounding ECM regarding the surface topography; the cell body can respond to pharmaceutical drugs, and also strengthen the power of differentiation [40]. It also provides structural cues that more accurately represent properties of natural tissue and regulate cell behavior, including the adhesion, expansion, and differentiation of various cell types. We can observe the NIH/3T3 cells cultured on the 2D surface of alginate/chitin hydrogel adhered strongly on day 3. The cells were well-grown with polygonal morphology and had shown extended cytoskeleton and dendrites-like structure. Cells were uniformly distributed and proliferated well on the surface. Notably, the cell body in 2D looks flattened shape and abnormal polarization which can be rarely observed on the 2D surface of alginate/chitin hydrogel. The result indicates that the composite has strong biocompatibility and cell adhesive capacity, which are in agreement with our previously reported work [27]. The fluorescence microscopy images of encapsulated NIH/3T3 cells within the cultured alginate/chitin of 3D microcapsules can be observed in Figure 9. The cells were well-proliferated and distributed relatively similar to the cells cultured in a 2D structure. The proliferation of cells was not affected in the microcapsules, but actin green was sporadically visualized, which occurred after structural deformation of hydrogel with regular washing of PBS during the staining stage. Importantly, the porous hydrogel, enriched with nanofibrils that mimic the physical features of ECM exhibited favorable microenvironments for cell growth and spreading. The cells observed with the composite hydrogel were densely packed together with faltering of the cell body, cytoskeleton webbing, and multi-directional stellate spreading with substantial development of the filamentous cytoskeleton. All these features indicated that the enhanced promotion of cell attachment and proliferation was achieved in CSMs. It is well-known that the spongy microsphere hydrogel has both cationic and anionic charges that influence protein interaction, biomolecule diffusion, and biological activity [41]. The electrostatic interactions and formation of intra- or intermolecular bonds generate the recognition sites for cell adhesion; as a result, more filopodia can be observed on CSMs. These results suggested that the alginate/chitin composite hydrogel is cyto-compatible with fibroblast cells.

## 4. Conclusions

In conclusion, we have developed bioinspired core–shell microcapsules of alginate/chitin for the core and alginate layer of the shell. The microcapsules possessed a very uniform and regular core–size structure under optimized conditions of alginate/chitin at a composition ratio of 60/40, feed rate of 3 mL/h, and applied voltage of 5 kV. The chitin nanofibrils in the microcapsule enhanced mechanical strength and maintained stability under physiological conditions. The cytocompatibility of the core–shell microcapsule was evaluated with the encapsulation of NIH/3T3 fibroblasts. It was found that the mechanically dynamic core–shell microcapsules promoted NIH/3T3 fibroblasts with 96% cell viability over day 11. Our finding supports further use of cell encapsulated core–shell microcapsules as highly efficient cell regenerative and cell delivery biomaterials for cell transplantation in wound healing and tissue engineering and the concept may provide great promise for many other biomedical applications.

## Supplementary Materials

The following supporting information can be downloaded at: https://www.mdpi.com/article/10.3390/nano13172470/s1, Figure S1. Morphology of core-shell microcapsules at different composition. Optical images of microcapsules with different ratios of alginate/chitin at the core, (a) 90/10, (b) 80/20, (c) 60/40. Shell composition was 1% *w*/*v* of alginate and flow rate of 3 mL/h at applied voltage 5 kV. Short view scale bar = 200 µm; Figure S2. Morphology of core-shell microcapsules at different flow rates. Optical images of microcapsules (alginate/chitin (60/40)) at applied voltage of 5 kV with flow rate of (a) 1 mL/h, (b) 3 mL/h, and (c) 6 mL/h. Shell composition was 1% *w*/*v* of alginate. Short view scale bar = 200 µm; Figure S3. Morphology of core-shell microcapsules at different voltages. Optical images of microcapsules with alginate/chitin (60/40) with flow rate of 3 mL/h at different applied voltage (a) 1 kV, (b) 5 kV, and (c) 8 kV. Shell composition was 1% *w*/*v* of alginate. Short view scale bar = 200 µm; Figure S4. In vitro cell culture with monolithic microcapsules. Microscopy images of NIH/3T3 cells encapsulated alginate microcapsules at day 9.
