# Supplementary material for: Chitin Nanofibrils Enabled Core–Shell Microcapsules of Alginate Hydrogel
Source: Nanomaterials (Basel). 2023 Sep 1;13(17):2470. doi: 10.3390/nano13172470 (PMC10489914; doi:10.3390/nano13172470)
Supplement: Supplementary file 1 [file nanomaterials-13-02470-s001.zip › nanomaterials-2579748-supplementary.pdf]

Supplementary information

Chitin Nanofibrils Enabled Core–Shell Microcapsules of Alginate Hydrogel

Thakur Sapkota [1,2,†], Bishnu Kumar Shrestha [1,†], Sita Shrestha [1] and Narayan Bhattarai [1,2,*]

[1] Department of Chemical, Biological, and Bioengineering, North Carolina A&T State University,
Greensboro, NC 27411, USA; tsapkota@aggies.ncat.edu (T.S.); bishnuaampipal@hotmail.com (B.K.S.); sshrestha2@ncat.edu (S.S.)

[2] Department of Applied Science and Technology, North Carolina A&T State University, Greensboro, NC 27411, USA

* Correspondence: nbhattar@ncat.edu

† These authors contributed equally to this work.

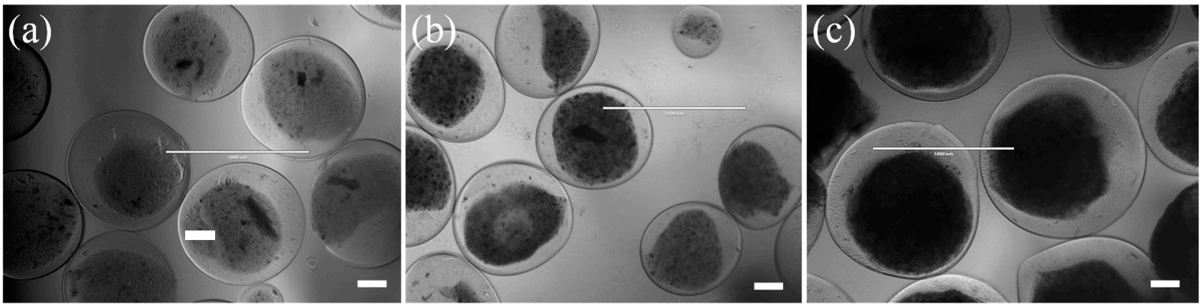

Figure S1. Morphology of core-shell microcapsules at different composition. Optical images of microcapsules with different ratios of alginate/chitin at the core, (a) 90/10, (b) 80/20, (c) 60/40. Shell composition was 1% w/v of alginate and flow rate of 3 mL/h at applied voltage 5 kV. Short view scale bar =200 µm.

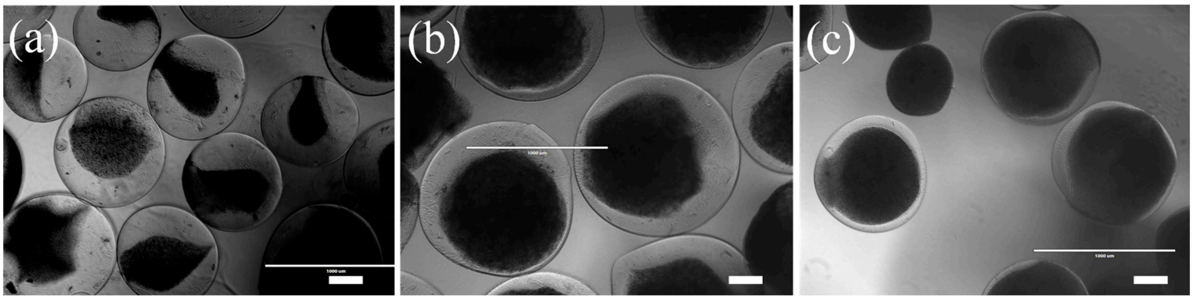

Figure S2. Morphology of core-shell microcapsules at different flow rates. Optical images of microcapsules (alginate/chitin (60/40)) at applied voltage of 5 kV with flow rate of (a) 1 mL/h, (b) 3 mL/h, and (c) 6 mL/h. Shell composition was 1% w/v of alginate. Short view scale bar =200 µm.

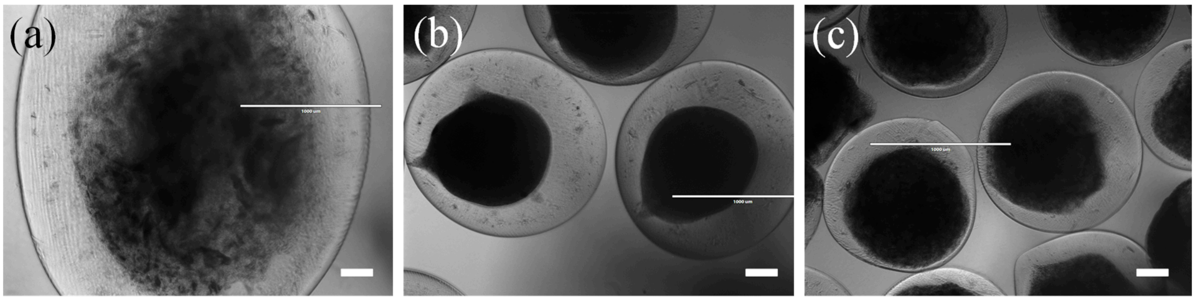

Figure S3. Morphology of core-shell microcapsules at different voltages. Optical images of microcapsules of alginate/chitin (60/40) with flow rate of 3 mL/h at different applied voltage (a) 1 kV, (b) 5 kV, and (c) 8 kV. Shell composition was 1% w/v of alginate. Short view scale bar = 200 μm.

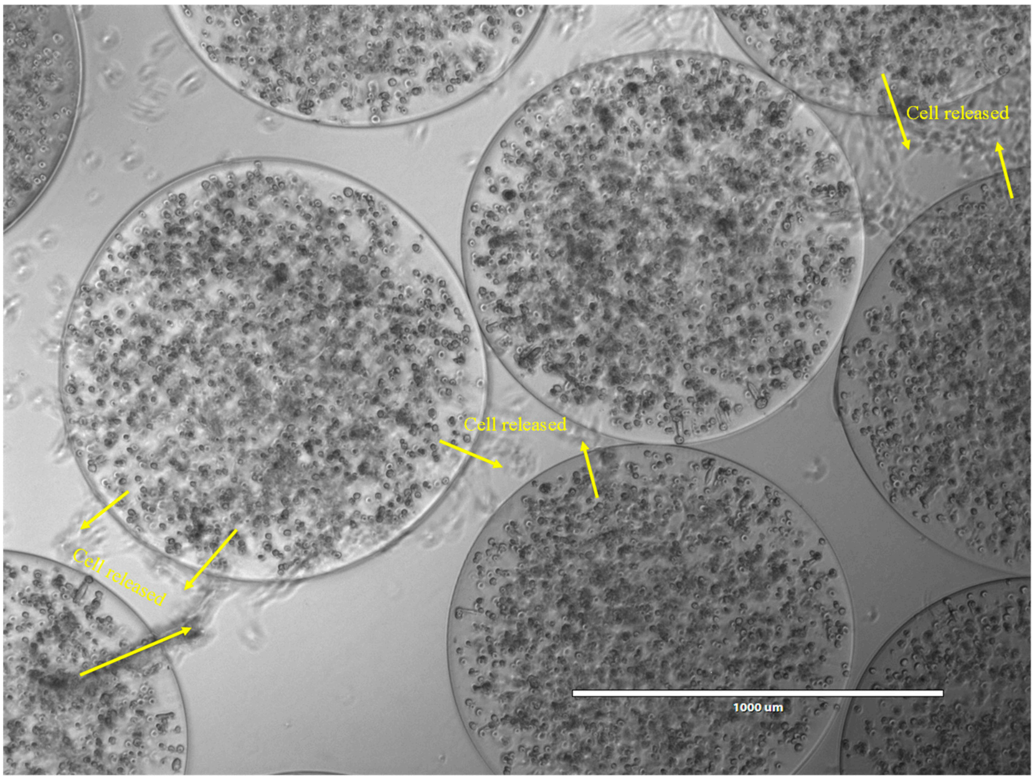

Figure S4. In vitro cell culture with monolithic microcapsules. Microscopy images of NIH/3T3 cells encapsulated alginate microcapsules at day 9.
